# Physicians’ Perspectives on AI in Clinical Decision Support Systems: Interview Study of the CURATE.AI Personalized Dose Optimization Platform

**DOI:** 10.2196/48476

**Published:** 2023-10-30

**Authors:** Smrithi Vijayakumar, V Vien Lee, Qiao Ying Leong, Soo Jung Hong, Agata Blasiak, Dean Ho

**Affiliations:** 1 The N.1 Institute for Health, National University of Singapore Singapore Singapore; 2 Department of Communications and New Media, National University of Singapore Singapore Singapore; 3 Department of Biomedical Engineering, National University of Singapore Singapore Singapore; 4 The Institute for Digital Medicine (WisDM), Yong Loo Lin School of Medicine, National University of Singapore Singapore Singapore; 5 Department of Pharmacology, Yong Loo Lin School of Medicine, National University of Singapore Singapore Singapore

**Keywords:** artificial intelligence, AI, clinical decision support system, CDSS, adoption, perception, decision support, acceptance, perception, perspective, perspectives, opinion, attitude, qualitative, focus, interview, interviews

## Abstract

**Background:**

Physicians play a key role in integrating new clinical technology into care practices through user feedback and growth propositions to developers of the technology. As physicians are stakeholders involved through the technology iteration process, understanding their roles as users can provide nuanced insights into the workings of these technologies that are being explored. Therefore, understanding physicians’ perceptions can be critical toward clinical validation, implementation, and downstream adoption. Given the increasing prevalence of clinical decision support systems (CDSSs), there remains a need to gain an in-depth understanding of physicians’ perceptions and expectations toward their downstream implementation. This paper explores physicians’ perceptions of integrating CURATE.AI, a novel artificial intelligence (AI)–based and clinical stage personalized dosing CDSSs, into clinical practice.

**Objective:**

This study aims to understand physicians’ perspectives of integrating CURATE.AI for clinical work and to gather insights on considerations of the implementation of AI-based CDSS tools.

**Methods:**

A total of 12 participants completed semistructured interviews examining their knowledge, experience, attitudes, risks, and future course of the personalized combination therapy dosing platform, CURATE.AI. Interviews were audio recorded, transcribed verbatim, and coded manually. The data were thematically analyzed.

**Results:**

Overall, 3 broad themes and 9 subthemes were identified through thematic analysis. The themes covered considerations that physicians perceived as significant across various stages of new technology development, including trial, clinical implementation, and mass adoption.

**Conclusions:**

The study laid out the various ways physicians interpreted an AI-based personalized dosing CDSS, CURATE.AI, for their clinical practice. The research pointed out that physicians’ expectations during the different stages of technology exploration can be nuanced and layered with expectations of implementation that are relevant for technology developers and researchers.

## Introduction

### Background

A clinical decision support system (CDSS) is a widely established tool to enhance health system efficiency. Administered through electronic medical records and other computerized workflows, a CDSS has been established to improve clinical practices [1]. For example, patient health outcomes from treatment presented through visual prebuilt reports can provide insights to physicians regarding patterns of care and patient responses, thereby improving the experience of treatment provision.

Aimed at enhancing ease of decision-making and reducing medical errors, a CDSS covers a range of tools used independently or in combination. CDSS types commonly include informational support (eg, access to information on clinical condition and patient data), patient insight support (eg, visual reports of patient history and customized support such as drug-drug interactions for specific patients), and personalized clinical data support (such as computational medicine based on specific patient data) [2].

The incorporation of artificial intelligence (AI) further expands the capabilities of CDSS and elevates its efficiency. Personalized medicine is a domain of health care that has benefited from AI’s capabilities of advanced data analytics for diagnosis, prognosis, and customized care strategies. Leveraging sophisticated computation and inference mechanisms, AI in personalized medicine has a potential to be impactful in terms of disease management, reducing adverse events, and containing health care costs in the long run [3].

Defined as care customized to predicted response or risk of disease in the patient, personalized medicine is considered to improve treatment pathways for patients by improving the accuracy of diagnosis and tailoring treatment plans that can offer enhanced health outcomes [4]. Drug selection, drug optimization, treatment regimen, prediction of treatments, and response outcomes are key areas of research in personalized health that have demonstrated the potential to improve treatment pathways for patients. For example, AI can be used to understand the binding properties of genomic sequences to predict the sequence specificity of DNA- and RNA-binding proteins [5]. Genomic profiling using AI has similarly shown to provide improved treatment pathways for patients with cancer [6]. CURATE.AI is an AI-derived, personalized medicine platform that offers physicians a support in making dosing decisions tailored to each patient based on individual patients’ profiles. CURATE.AI maps the relationship between an intervention intensity (input) and a phenotypic result (output) for an individual based exclusively on that individual’s data for decisions on that individual’s dosing strategy only. As the individual’s health status or treatment changes, for example, as disease progressesor recesses, new drugs are added, and medical interventions are administered, the CURATE.AI profile also changes, which is recalibrated for the most optimal care through the course of treatment [7]. CURATE.AI has been clinically assessed across multiple indications, ranging from oncology to immunosuppression. These have included prospective, interventional studies, as well as retrospective analysis studies [8-15]. It has also been explored in the domain of personalized cognitive training in healthy individuals [16]. Several prospective interventional studies are also ongoing or being cleared for initiation [17-23].

**Figure 1:**
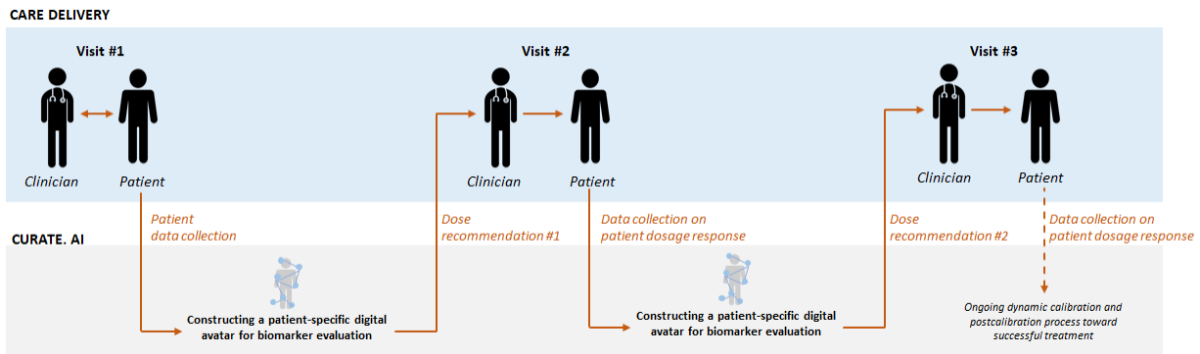
CURATE.AI clinical implementation workflow. The arrows indicate the flow of the data.

CURATE.AI differs substantially from the current community of CDSS platforms. For example, it does not use population-derived big data to train algorithms for the treatment of each subsequent patient. Instead, it uses only a patient’s own data to mediate their own treatment. These data are based on calibrating a patient’s clinical response (eg, clinically actionable biomarker dynamics) to variable dosing. As such, unless there are preexisting data for each patient that correlate multilevel drug dosing with corresponding biomarker levels for each dose, there is typically no starting data set for CURATE.AI-guided treatment. Therefore, CURATE.AI-based intervention relies on physician engagement at the very beginning of its implementation road map—the building of a patient-specific small data set based on modulated dosing and biomarker readings. This information is then used to construct a patient-specific digital avatar. This avatar provides actionable dosing guidance, and the subsequent measurements of a patient’s response to treatment drive the evolution of this avatar to continuously recommend downstream dosing guidance. This guidance can potentially result in dosing modulation during the course of treatment. Another key differentiator of CURATE.AI is that its dose recommendations, similar to its calibration process, can be dynamic. Therefore, a longitudinal dose modification and the corresponding evolution of the digital avatar are likely. This further relies on physicians’ engagement during the intervention process. These factors, defined by a CDSS that is based on longitudinally modulated patient dosing, provide insight into the rationale of this study, as sustained physician engagement is a cornerstone of CURATE.AI implementation (Figure 1).

In terms of clinical implementation of CURATE.AI, the key goal is to develop a platform that by design is in the best position to overcome *pilotitis*, an inability to progress past the pilot trial, and address the issues such as clinical acceptability; interoperability with the existing systems; and alignment with the prevailing privacy, safety, and regulatory frameworks, among others [24]. Therefore, CURATE.AI benefits greatly from including the stakeholders’ and physicians’ views at the tool development stage.

### Objectives

In the context of AI-based personalized medicine, physician acceptance and sustained use remain a continuous challenge although its promise and benefits are widely recognized [25]. Successful real-world application depends on clinical workflows [26] and the scope of physicians to rely on such tools to improve their current practice [27]. Physicians’ intent and expectations remain a key human factor that influences outcomes in clinical trials as well as sustained use of CDSS tools [28]. Physician endorsement and acceptance [27], specifically in the initial exploratory stages of new technologies such as in clinical trials, can facilitate meaningful integration into work practices [28]. Understanding the workload of decision-making from the physicians’ perspective, the potential of new technologies to improve accuracy of medical recommendations while at the same time foregrounding patient safety can be key to charting implementation goals and milestones [29].

Furthermore, for transition to clinical practice, it is vital to enable continued evidence building, which in turn benefits from understanding implementation challenges among the stakeholders [30]. Although physicians, in general, report a positive attitude toward the potential of CDSSs for transforming medical practice [28], resistance toward the newer capabilities of AI such as in personalized medicine can renew discussion on patient safety concerns, clinical evidence, and greater technology design involvement on the part of health professionals [27,29,31]. This can similarly influence the levels of acceptance and introduce barriers in deployment [30]. Furthermore, technology hesitancies not only hinder uptake but also reduce the scope to produce evidence from sustained use [32]. Misunderstandings and mistrust with support tools also reduce the opportunity to realize the potential from a complete use of such tools for clinical decision-making [33].

The understanding and reaction of physicians to new clinical tools are therefore crucial factors to enable clinical integration and ensure downstream adoption [30,32,34]. To date, physicians’ perspectives in emerging technologies are a relatively underexplored domain and can be beneficial to explore to enable the discussion of provider-aligned implementation of new technologies [35].

In the context of CURATE.AI, its expanding clinical applications, such as in combination products and medical software, imply new opportunities and trajectories that alter care formats [16]. With physicians playing a key role in integrating such tools into care practices, they can provide impactful user feedback and growth propositions to developers of the technology [36]. As stakeholders are involved in the process of its iterations [18], understanding a physician as a user can provide nuanced insights into the workings of CURATE.AI and broadly AI-based CDSS tools. This is also a critical factor that can be relevant to enable desired adoption [27,37], a discussion often overlooked by the technology developers.

This study accordingly gathers insights of physicians through their understanding of integrating CURATE.AI for their clinical work. Drawing these perspectives based on physicians’ involvement with the personalized dosing platform, the study outlines key considerations that matter for AI-based CDSS implementation, covering aspects of trial, clinical, and technology adoption considerations.

## Methods

### Overview

This study adopted an exploratory qualitative approach. Given the relatively sparse research on physicians’ attitudes and behavior toward AI-based CDSS implementation, a qualitative approach was used as it enables eliciting user views in a relatively unrestrained manner. Similarly, qualitative methods hold the potential to bring forth insights on various considerations that go into contexts [28], which can be valuable in terms of gaining nuanced insights on CDSS.

### Ethical Considerations

The study was approved by the National University of Singapore Institute Review Board (#LS-20-140E). Interviews were conducted either in person or were web based. Participants provided written informed consent before participating in the interviews. No reimbursement was provided. Data was stored in secured folders and accessed by researchers who were part of the study. All data used for publication is anonymized.

### Recruitment and Procedure

The inclusion criterion for purposeful sampling of the expert interviews was medical professionals, including physicians and medical students who were familiar with CURATE.AI. All recruited participants were from the National University Hospital or the National University of Singapore. They were contacted via email to understand their interest to participate in the study. Before the interview, each participant was informed about the purpose of the study, the recruitment criteria, the interview process including reasons and interest in the research topic, and the right to withdraw at any point throughout or after the study. Each participant signed a consent form before being interviewed. All interviews were conducted by 2 female interviewers (SV and QYL) trained in qualitative research based on a semistructured interview guide covering topics on knowledge, uncertainties, risks, and implementation of CDSSs. Information on the medical field of the participants and years of practice was collected as basic demographic information in the interviews. As the central discussion in the interviews was to bring up participants’ understanding and implementation considerations of CURATE.AI, greater focus was placed on questions pertaining to the same. All interviews were audio recorded and transcribed verbatim. Only the researchers who were part of the study were present during the interview. No repeat interviews were conducted. Data were discussed among researchers to confirm data saturation. The interview guidelines are presented in Textbox 1.

**Interview topic guide.**

**Understanding of CURATE.AI**

1. Knowledge of CURATE.AI
2. Confidence and uncertainty of the use of CURATE.AI in a clinical setting
3. Concerns regarding privacy and trust in the use of CURATE.AI in a clinical setting
4. Assumed level of confidence, uncertainty, and trust in the use of CURATE.AI held by the patients
5. Determining factors that promote the use of CURATE.AI
6. Additional advantageous or adverse factors that might affect the use of CURATE.AI

**Adopting CURATE.AI as a clinical decision support system**

1. Definitions of successful treatment
2. Perceptions of incorporating CURATE.AI into clinical settings and the standard of care
3. Benefits of adopting CURATE.AI in clinical care
4. Barriers in adopting CURATE.AI in clinical care

### Data Analysis

In line with the interpretive tradition in qualitative research, data were analyzed thematically, condensing meanings based on participant descriptions and researcher interpretations. This method of analysis, also called the process of meaning condensation, involves identifying ideas emerging from the text to make sense of descriptions analytically [38,39]. Data analysis began with the reading and rereading of the transcripts for open coding, that is, descriptively labeling the data. This was performed manually by identifying words, phrases, and sentences that conveyed specific ideas. This was followed by gathering these descriptive labels into potential themes and collating relevant data under each broader theme, a step referred to as axial coding. Subsequently, the data were further examined to understand how themes worked in relation to each other, refining the specifics of each theme and grouping them further based on emerging insights, a step called selective coding [38,39]. Assertions were drawn from the data following data saturation. All coding was performed manually by 3 researchers (SV, VVL, and QYL), part of the study team, all of whom were trained in qualitative research. The guidelines in Consolidated Criteria for Reporting Qualitative Research [40] have been adhered to.

## Results

### Participant Characteristics

A total of 21 participants were invited to participate in the study by email. Of these, 2 (10%) participants declined and 6 (29%) participants did not respond to the recruitment email. A total of 12 interviews were conducted with interviewees—consultants (including associate and senior) and 2 medical students—covering specialties such as internal medicine, oncology, gastroenterology, general surgery, cardiology, neurology, hematology, and ophthalmology. As CURATE.AI is indication agnostic and can be applied to any medical indication, independent of the setting of the physician, we covered a range of medical specialties. Furthermore, to gain diverse perspectives of CURATE.AI in terms of its implementation, we interviewed physicians and medical students who had varied levels of engagement with CURATE.AI (ie, the data included interviews with participants who were part of the initial and ongoing clinical trials and discussions of CURATE.AI). In total, 11 interviews were conducted on the web and 1 in person based on the convenience of the participants. Interviews lasted between 16 and 56 minutes.

### Interview Data

A total of 3 themes and 9 subthemes were identified in the data based on data coding. Textbox 2 captures the themes and the mentions for each theme. The 3 themes were trial considerations, clinical considerations, and technology adoption considerations. Trial considerations covered ideas pertaining to piloting of CURATE.AI and aspects pertinent to building evidence before CURATE.AI’s clinical adoption. Clinical considerations underscored the aspects of relevance in using CURATE.AI within the context of the clinic, and the technology adoption considerations emphasized the factors essential to enable the broader implementation of CURATE.AI. Although aspects within each theme can be relevant across themes, they are categorized based on their closest relevance within the stages of trial, clinical, and broad adoption.

**Themes and subthemes.**

**Trial considerations**

1. Attitude toward CURATE.AI
  - Improved drug predictability
  - Personalized profiling
  - Potential to transform medical practice
2. Evidence and clinical decision-making control
  - Level of evidence
  - Accuracy and reproducibility
3. Patient safety
  - No adverse effects
  - Physician’s final say
4. Trial data availability
  - Access to trial data
  - Access to treatment protocols

**Clinical considerations**

1. Method of CURATE.AI
  - New language of treatment
  - Negotiating the idea of
    Machine vs Physician
2. CURATE.AI and standard of care
  - Differentiating CURATE.AI
  - Establishing CURATE.AI step by step
3. Awareness and clinical integration
  - CURATE.AI as a concept of care
  - System to access info and data on CURATE.AI
  - Access to the CURATE.AI software

**Technology adoption considerations**

1. Preventing siloed functioning
  - Communication and interaction with relevant teams
  - Bringing together expertise
2. Idea of product realization in CURATE.AI
  - Clinically instinctive
  - Ease of use
  - Integrated use of CURATE.AI

### Trial Considerations

#### Attitude Toward CURATE.AI

Interviewees, including physicians and medical students, conveyed an overall positive attitude toward exploring the use of an AI-based platform, highlighting its potential to improve the predictability of the patient response to the treatment at a given intensity, which otherwise can be a challenge. Interviewee 4 shared the following:

I think for some drugs...there’s a lot of unpredictability. So the whole idea of CURATE.AI is to provide some sort of predictability to it...I think that’s the main advantage for it.

In that sense, physicians repeat the idea of CURATE.AI enabling a way that transforms current practice of care. As interviewee 9 expressed, “It’s something that has the potential to change the way we practice medicine.” Interviewees discussed the novelty of the idea in the unique advantage it brings in terms of drug dosing. Interviewee 3 elaborated as follows:

And what is interesting is the ability of CURATE.AI to design personalised profiles of patients using a biomarker of efficacy as an input parameter to be able to modulate doses. This is something that is relatively unique and has not been done before.

Interviewee 2 echoed a similar sentiment:

Something that used to be very difficult to do, now can be done by machine. Something that we don’t think can be done now, there’s a chance that it can be done.

#### Evidence and Clinical Decision-Making Control

However, interviewees’ openness to the technology came with caveats that were acknowledged equally important. These caveats were repeated across the board, highlighting the considerations interviewees perceived salient in the pilot testing that CURATE.AI was in at the time the interviews were conducted. Interviewee 3 highlighted, “And I think the most critical thing at this point of time, is the need to be able to show that the CURATE.AI platform can actually be applied in patients and is indeed predicting doses that are better or more appropriate for the patients.” Evidence through clinical trials therefore was underscored as a critical next step. As interviewee 6 stated, “So to build confidence, number one—need to look at the level of evidence right? And that’s why we are doing a clinical trial as a step of providing clinical evidence.” Building accurate and reproducible evidence in this manner emerged as key, as interviewees repeatedly emphasized the data-driven nature of technology adoption in clinical contexts. Interviewee 6 highlighted:

There’s an inherent concern about the accuracy or the reproducibility of the clinical decision support tool, before a widespread use would be possible. So hence, I think the key thing is just to generate good data, so that the clinician can be convinced.

Also stated as salient was the need to build evidence across regimens to improve physicians’ confidence. Interviewee 3 elaborated, “It [CURATE.AI evidence] needs to be established across different regimens, and most definitely we’ll have to run different trials in each regimen.”

Although building evidence emerged as a key consideration in the pilot stage of CURATE.AI trials, the interviewees highlighted the need to continue to be in charge of decision-making, suggesting that the role of a CDSS platform is to be assistive in clinical work. Interviewee 4 stated, “Firstly, the doctor needs to understand the basis [of CURATE.AI] and secondly, the doctor needs to make the final decision, [only] then it can be considered as CDSS, otherwise it can’t.” Underlying this was a sense of risk conveyed by the doctors. Despite acknowledging the promise of CURATE.AI, they preferred remaining cautious owing to possible clinical risks, as interviewee 4 highlighted, “Doctor’s having the final say helps.”

#### Patient Safety

Important in this journey of evidence building was to pay attention to the facets of patient safety in CURATE.AI’s capabilities. Interviewee 8 shared, “I think the greatest way of convincing people that you are on the right track is that you can show them that this method really reduces [clinical symptoms] safely and there are no side effects.” Therefore, evidence of efficacy was critically linked to patient safety. Patient safety and concerns of patient risk were tied back to the physician being in control, in that physicians conveyed their final say in decisions for the patients as a method of setting safeguards. As interviewee 1 elaborated, “I think there are safeguards in place like the clinicians having the final say about the dosing and then they are able to preset safety limits—the upper range and the lower range—so I think that helps to alleviate some of these concerns [risks].”

#### Trial Data Availability

In terms of envisioning widespread willingness to adopt the technology, interviewees underscored the need to have access to trial data to promote confidence and certainty among physicians. Physicians expressed that the lack of such access may hinder adoption and reduce confidence. As interviewee 1 stated, “the lacking part that maybe stopping doctors from using would be, number one, whether there is a full trial available so doctors will be more willing and be more convinced.” Envisioning this can be an important consideration especially as doctors have highlighted the difficulty in understanding the process and method outside of the trial context. Interviewee 8 elaborated as follows:

Within a trial, you actually have a protocol, which you follow. Outside the trial, it’s much more difficult to figure out why they are doing, what they are doing and why.

### Clinical Considerations

#### Method of CURATE.AI

As an altered method of decision-making by physicians, the assistance of CURATE.AI can mean changes in the treatment method and outcomes for both physicians and patients. Considering the introduction of CURATE.AI as a process, physicians have highlighted the need to learn and adjust to its assistance to ensure its clinical success. A revised dose recommendation based on CURATE.AI may represent a new treatment experience for both the patients and physicians. Patients’ understanding of the process therefore can be critical in enabling physicians to use the platform effectively. Interviewee 2 shared the following in this regard:

Someone [patient] is actually getting better, but tells you that there’s no difference [due to reduced drug dose recommendation] then you know, whether you trust the patient or not. I guess the patient will have to learn a certain kind of new language when it comes to this kind of machine treatment, machine-led treatment plan. So it’s a lot of new language to learn for both sides.

Interviewee 1 echoed a similar sentiment, highlighting that CURATE.AI’s novelty can impact physician-patient interaction as well as their perception of the treatment method. The question of machine-mediated and standard practice will likely be a constant consideration for the patients that physicians will need to face:

Think if I were to think about day-to-day interactions with patients. I think the concerns would be that it’s [CURATE.AI] a very, very new concept. It will then be a problem to them, to the very end, thinking about whether it is machine versus doctor kind of dosing.

#### CURATE.AI and Standard of Care

Interviewees expressed the need for CURATE.AI to differentiate itself in a way that makes its presence more efficacious for the patient than the standard of care. Interviewee 6 stated, “Getting evidence to convince people that – hey it is actually better than what normal people would do – it’s very important.” Marking itself as a method better than what is currently practiced was repeated as an idea with physicians underscoring the need for evidence to demonstrate this advantage. As interviewee 8 shared, “You need to have situations where CURATE.AI is obviously better than what we are doing now.” Although physicians strongly recommended this, in terms of establishing this, they encouraged a step-by-step approach in that building proof-of-concept is work in progress and needs to be managed realistically as highlighted by interviewee 8 that in terms of next steps for CURATE.AI, “I would say don’t try to do everything.”

#### Awareness and Clinical Integration

Physicians’ awareness was highlighted as vital in clinical integration. Novelty of the concept being a key reason, physicians identified a need to make the idea of AI in decision-making familiar among physicians to ensure its clinical adoption. Interviewee 1 shared, “Think first increasing awareness amongst clinicians [is important] because I think, at least from what I talk to my colleagues and doctors about, this concept of, maybe not just CURATE, but AI generally as a use within clinical settings is still relatively new.”

In envisioning clinical integration, interviewees recommended a system to be able to access clinical evidence and recommendations swiftly to improve physician confidence. Interviewee 1 elaborated, “We were talking to other doctors, so what we hear and [what] I personally think that there has to be a system - if you really want it to support doctor decision-making, there should be an interface whereby doctors can go onto it and get results quickly, at least within a stipulated timeframe.”

The emphasis on the system was to enable a more independent use of CURATE.AI that can help with ease in clinical adoption, as interviewee 1 explained further:

Because I think at this trial stage, CURATE. AI is still very much being manned by the CURATE. AI team so there isn’t an available public software that people can go into. So, if it can be made more easily accessible to doctors, I think that would help as well.

### Technology Adoption Considerations

#### Preventing Siloed Functioning

Interviewees recommended efficient collaboration across teams with varied expertise to be the method of implementation to adopt to ensure efficiency in clinical adoption and practice. Interviewee 4 shared why this can be critical, identifying collaboration is key to bring together expertise that cannot work separately:

They [engineering team] will run the data analysis and then they will tell me about the various methods for CURATE.AI. So mainly I provided the clinical advice, the clinical aspect, or to see how the data could be clinically relevant, and then they will, on their end, they will run the data analysis and see how we can work together to make it better.

The idea of collaboration was also highlighted as relevant in building and enhancing CURATE.AI. Physicians identified the need to bring together expertise from different groups to ensure comprehensiveness and to be able to build a more relevant final product. Interviewee 5 expressed the following:

So to learn from another work group, [that’s] the way you should go about building some of these things. Because it consists of people who are experts in their fields. So whether it’s a domain expert that looks at clinicians, who are experts in prescribing the drugs – they are the ones with the patients. Or technical people, who look at supporting the clinical domain experts. Or the science aspect, the actual validation crew or the people who actually do the validation on the scientific basis. They all need to come together, because you can’t run this in silos, right? And what will happen if you run it in silos, you will get what the silos product is.

Interviewee 12 echoed a similar sentiment, “Keep working, but make sure you don’t work in your own silo, make sure you work with a good collaborative partner, that is very important.”

The need for collaboration also covered efficient communication during implementation, wherein physicians indicated the need for different teams to come together for effective execution. As interviewee 11 shared the following:

The interaction with the AI team is critical, because we also need to relay the clinical findings, the toxicities that the patients have felt. So, finding a quick way to relay that information across and then for feedback is very important.

#### Idea of Product Realization in CURATE.AI

Built into the idea of technology adoption are considerations that physicians recommended to create capabilities that will enable the easier transition of CURATE.AI to mainstream care. Ease of use with minimal interaction with multiple teams at the point of delivery was a key facet physicians identified to make adoption simpler. Sharing an example to explain the idea interviewee 6 elaborated, “So if you imagine yourself as a service provider, either that you’re making an AI-related phone or a service ideally it should be instinctive, as easy, without too much interaction with the service provider, that would be ideal right?”

Similarly, ease of use is to extend to the actual use of the platform to enable sustained use of the platform and continued adoption. Interviewee 6 further expressed the following:

Usability and the ease of use. Like what I say, if it’s too much trouble, not instinctive, then you find that doctor would revert back to their old ways. So it needs to be easy to use and a doctor need to be able to feel confident using it. So I think those are important things for widespread use.

Beyond the idea of a simplified and an easy-to-use platform, physicians also identified its compatibility and integration into practice as important aspects to consider to facilitate a seamless use of the platform. Conveying it through an instance, interviewee 8 shared the following:

Not just simplify, but to integrate. So, in other words, if you have this electronic prescription system, you should put CURATE.AI into it and say, “Here’s an app,” which automatically switches on and it will only give you advice when it is pertinent. So, could have a little board there saying, “Oh, I see you are prescribing anti-hypertensive drugs, may I help you? I will optimise the patient’s dose.” Okay? Then, if you say yes, then the computer says, “Okay, I note that this patient is on this, this, this and this drug, okay? Is the blood pressure control optimal? Yes or No?”. If you say, “Yes,” then the computer says, “Great! Carry on,” or it might give you some other advice. If you say, “no,” then you ask, “Is it too high, too low?” and then the computer gives you a suggestion.

## Discussion

### Principal Findings

This study identified physicians’ perceptions of AI-based CDSS through the context of a personalized drug dosing platform, CURATE.AI. The findings demonstrated the various considerations physicians articulate in the idea of using CURATE.AI in their practice. In general, physicians expressed the promise of CURATE.AI in transforming and elevating the standard practice. However, physicians perceived several crucial considerations relevant for success of CURATE.AI as it progresses through the stages of trials, clinical integration, and eventual adoption in mainstream care.

Aligned with the idea that a CDSS holds potential to improve patient safety and prevent human error [41], trial considerations about CURATE.AI were one of the foremost aspects covered by physicians. These aspects linked to the early stages of technology development covered strategies to enable CURATE.AI’s successful progression to subsequent stages. Largely built on a positive narrative, physicians shared a technology-embracing attitude that conveyed the potential of a CDSS to transform medical practice for the better. However, built within the optimism, there was a need for the tool to be supported by solid and sound evidence of its effectiveness. Validating a CDSS is a key initial step in CDSS development and can play a crucial role in physician acceptance as altered treatment mechanisms can result in differential patient outcomes [42-44]. Physicians, in this regard, described evidence building as a first and necessary step to envisioning an effective final product.

Furthermore, the difference in patient outcomes in different medical interventional contexts means that trials must accommodate for this variation in patient experience to prevent misjudgment of trial data [45,46]. Physicians acknowledged this, conveying the need for evidence to cover an expanse of treatment specialties and regimens to be able to foreground patient safety in the development of AI-based CDSS platforms such as CURATE.AI.

Weaved into the idea of patient safety was also the need for the platform to ensure the absence of side effects or adverse effects. The concern of patient safety is often cited as a key setback in CDSS implementation, as the reliance on technology can alter physician-patient communication and relationship [32]. For instance, the physician’s reliance on technology for assistance can be seen as a hindrance as they also manage patients’ desire for having a choice if AI will be used by the physicians for their care [47]. Hence, in terms of patient-physician relationship, the physicians may feel a sense of reduction in autonomy and increase in uncertainty when the technology is driving the decisions [48,49]. Physicians accordingly linked patient safety to their need to make the final call with a CDSS working only as a supportive mechanism and their decisions of recommendation agreement or disagreement being the final medical suggestion to convey to the patient.

Toward clinical integration, physicians conveyed the need to negotiate the difference in the method of CURATE.AI and standard practice in their medical communication with the patients. The presence of CDSS tools can mean a transformed health care experience for both the physicians and patients [50,51]. Numerous tools in the domains of diagnosis, prognosis, and personalized treatment pathways have underscored the possibility of better health outcomes through renewed treatment protocols [7]. For instance, in the area of diagnosis, an evaluation of a deep learning approach for electrocardiogram analysis reports the ability to categorize a wide range of arrhythmias to lower or prevent misdiagnosis [52]. Similarly, research in prognosis demonstrates the potential of deep learning models in forecasting disease outcomes to explore possible treatment scenarios [53], and frequent pattern mining enables targeted therapy in lung cancer treatments [54].

However, most health technology transformations introduce a variation in medical interaction, including the understanding of treatment protocol and success measures [55]. In this regard, physicians described the need to both understand the altered method themselves as well as translate that to the patients, resulting in a negotiation of what is better (comparing the standard of care with new technology-assisted dosing). Physician training is a recommended step to enhance the efficient use of CDSS particularly in terms of the physicians’ understanding of the tool [56]. Explainability perceived by physicians (ie, the ability of a user to explain how the system reached a decision [57]) often facilitates efficient communication, use, and trustworthiness among both physicians and patients [58].

Furthermore, patients’ resistance to new technologies emerging from technology anxiety is reported to affect their adoption and use and can lead to negative consequences [59]. The resistance often stems from the unfamiliarity, newness, and differential experience of the care process owing to the presence of technology [60]. Physicians accordingly highlighted the need for a better understanding of the language of CDSS both on the part of the patients as well as physicians to avert risks in communication and practice.

Although physicians expressed their responsibility to convey the strength of a CDSS to patients, their ability to do so in the clinical context was yet again a factor tied to the available evidence. In this case, establishing CURATE.AI as a more efficient method equivalent to the standard of care was critical. Introduction of technology is often cited to induce a sense of discomfort and lesser control in patients who are new or unfamiliar with new technologies [59]. Therefore, physicians take up the responsibility to vouch for the effectiveness of CURATE.AI. Building physician confidence through clinical evidence as well as access to data can be crucial in the clinical integration of the support tool [61-63].

In envisioning an AI-based CDSS for adoption in mainstream care, physicians expressed the importance of early strategizing. For example, the ability to generalize AI algorithms at an early point can enable creating a more efficient road map for AI-based tool implementation. Recent research on personalized AI approaches in oncology (such as personalized medicine tools explored for gliomas) discusses this implementation barrier where to date, the used AI has largely been trained on smaller populations, preventing applicability for groups that may be heterogeneous [64].

Similarly, in terms of usability of technology, physicians relayed that clear goals of the technology coupled with a practice of collaborative functioning among implementing teams can enable a faster integration of AI-based CDSS tools into care practices.

Usability is often cited as an important factor to consider in CDSS implementation [65]. For instance, the ability of users to quickly learn the technology, remain error free, run efficiently, and to be user friendly are key attributes often linked to success in implementing decision support tools [65]. Physicians explained why it is important to consider this in the early stages of CURATE.AI’s development.

Furthermore, for the support tool to be clinically instinctive and seamless, technology needs to have evolved through iterations as well as through trial-based evidence. A simplified and integrated feel to the support tool therefore was a key preference in terms of technology adoption for the physicians, an end goal that is accomplished through the development cycle of the support tool. Furthermore, ease of use is also tied to the safety and prevention of adverse events from the use of such tools [66], an additional advantage the physicians articulated.

Clinical support tool effectiveness has often been tied to deployment approaches, and embedding support tools as part of the wider medical ecosystem has been cited to increase effectiveness of implementation [31]. Placing a CDSS as part of a wider community with multiple stakeholders drawing from diverse expertise is perceived as a necessary technology adoption strategy [31] in both design as well as use of the tool. Physicians expressed their preference for an open and collaborative approach in explaining a way forward for CURATE.AI.

The combination of diverse expertise with responsibilities of implementation aligned to skill brings forth the efficiency needed for effective implementation [31]. Physicians stated that such an approach would also support necessary conversations among relevant teams to facilitate knowledge flow as well as insights into effective designing and implementation. Weaving stakeholders such as the physicians into the process of tool development and implementation can also bring about a sense of involvement and accountability rather than a mere acceptance of a tool they have not contributed to. This can affect motivation and willingness to adopt [27,30,31].

To further understand the integration of new technologies, the Consolidated Framework for Implementation Research (CFIR) provides a helpful model for efficient incorporation of new technology in health underscoring key areas that matter for implementation [67]. The CFIR framework offers a way to outline enablers and barriers to delineate domains of implementation that can be tailored and adapted to facilitate efficient adoption of innovation [68]. Key domains include the nature of intervention (eg, adaptability, trialability, complexity, and design quality), outer setting (eg, patient needs and resources, cosmopolitanism, peer pressure, and external policy and incentives), inner setting (eg, structural characteristics, networks and communications, and culture), characteristics of individuals (eg, knowledge and beliefs about the intervention, self-efficacy, individual stage of change, individual identification with organization, and other personal attributes), and process (eg, planning, engaging, and executing) [69]. Mapping our findings to the CFIR in Table 1, we present physician insights as strategies that can facilitate the adoption of CURATE.AI among physicians.

**Table 1:** Mapping physician perspectives of CURATE.AI to Consolidated Framework for Implementation Research (CFIR) domains.

CFIR domain and relevant constructs		Physician insights to facilitate CURATE.AI implementation
**Intervention characteristics**		
	Evidence strength and quality	Establishing satisfactory levels of evidence for the adoption of CURATE.AI
	Relative advantage	Improved drug predictability using CURATE.AI vis-a-vis standard of care
	Adaptability	Accuracy and reproducibility of CURATE.AI
	Complexity	Personalized profiling accomplished through CURATE.AI CURATE.AI’s potential to transform medical practice
**Outer setting**		
	Patient needs and resources	No adverse effects in the use of CURATE. AI Physician’s final say in CURATE.AI-based treatment
**Inner setting**		
	Structural characteristics	Physicians’ access to trial data Physicians’ access to treatment protocols
	Networks and communications culture	Communication and interaction with relevant teams before and during CURATE.AI clinical implementation
	Implementation climate Readiness for implementation	Bringing together expertise to facilitate conversation, familiarity, and ease of implementation
**Characteristics of individuals**		
	Knowledge and beliefs about the intervention	Introducing and familiarizing physicians with the new language of treatment and negotiation idea of machine vs physician
**Process**		
	Planning	Differentiating CURATE.AI through its potential for improved care
	Engaging	Establishing CURATE.AI as a concept of care among physicians Enabling a step-by-step understanding of CURATE.AI
	Executing	Ensuring the presence of systems to access info and data on CURATE.AI Enabling an easy access to the CURATE.AI software
	Reflecting and evaluating	Evaluating the potential of CURATE.AI to be clinically instinctive Understanding ease of use and implementing course corrections Aiming for an integrated use of CURATE.AI in health care

Understanding implementation among physicians is a key factor to note the expectations of users especially in the relatively newer domain of an AI-based CDSS. Physicians as users of the technology can determine the eventual integration of new technologies into mainstream practice. Gathering perspectives of physicians in this regard is valuable as it situates technology within the context of the human actor [70]. For instance, our study identified the notion of patient safety and evidence building as crucial to adoption, where access to evidence can make a difference in physicians’ attitudes and adoption. Our results also contribute to the growing body of evidence on human-technology interaction that acknowledges the influence of social (eg, structure of the organization); psychological (eg, attitude toward technology); and cognitive characteristics (eg, biases of users) on user adoption, interaction, and sustained use of new technologies [58,71]. For example, physicians highlighted the need to get new technologies to demonstrate greater efficiency to enable easier acceptance of the technology.

### Limitations

As the goal of this study was to understand broadly the attitudes of physicians toward an AI-based CDSS through the case of CURATE.AI, physicians with different levels of engagement with the support tool were recruited. This was to enable a diverse perspective that attempted to capture the overall perception of the idea of an AI-based CDSS. As a varying group of physicians was included, a systematic or longitudinal CDSS experience among physicians was not covered. Furthermore, as purposeful sampling was used, it is possible that the recruited population was biased toward having a positive outlook on CURATE.AI. This could also be a reason for the observed absence of an association of the experience of physicians and their inclination to adopt personalized medicine. Hence, although our findings provide insights on personalized medicine implementation, it is important for future research to conduct more context-specific explorations. Exploring the experience of a CDSS longitudinally for a specific condition can add meaning in terms of nuances. This can be important especially because medical interventional contexts can vary significantly [72]. Such explorations can also shed light on complexities in design relevant to the medical condition, patient progress, safety, risks and uncertainties, and other implementation aspects [73]. Furthermore, this study covers the breadth of the entire cycle of CDSS development, including the phases of trial, clinical integration, and broad adoption and sustenance. This meant that the various stages are not dealt with in depth, and there remains scope for further discussion under each phase. This in-depth examination can be significant in improving current explorations and providing guidance in future efforts, including refining practices for better outcomes.

Another limitation is the possible limited generalizability of the findings as interviewee responses are likely to be tied to the specifics of Singapore health care system, the exposure to innovation, and the embedded attitudes to technological innovation potentially shaped by Singapore’s strategy for AI in health care [74].

### Conclusions

The study reported in this paper identified key factors that are relevant to physicians in the idea of an AI-based CDSS. Although physicians lay out numerous factors to consider in the different phases a CDSS tool goes through, physicians are generally open to the idea of new technology in advancing care practices. Evidence, patient safety, data availability, awareness, and collaborative functioning are key aspects that define technology adoption to physicians. Although these aspects outline the broader contours of technology adoption, the study has also delineated the nuances that go into these aspects, such as the nature of evidence building required, what matters for patient safety, the method to make data available, and preferences of awareness and collaboration required for clinical integration and sustained use. An AI-based CDSS such as CURATE AI represents a paradigm shift in health care and is set to redefine and enhance current medical practice [7,75]. Evidence on its potential to support physicians has also increased in the past decades. Continued research highlighting physicians’ role and patient attitudes [76,77] involvement can be valuable in reaching higher potential of a CDSS to support and transform clinical decision-making for the better.

## Abbreviations

AI: artificial intelligence
CDSS: clinical decision support system
CFIR: Consolidated Framework for Implementation Research

## Data Availability

The data sets used or analyzed during this study are available from the corresponding author upon reasonable request.
